# Impact of Maladaptive Daydreaming on Grade Point Average (GPA) and the Association Between Maladaptive Daydreaming and Generalized Anxiety Disorder (GAD)

**DOI:** 10.7759/cureus.10776

**Published:** 2020-10-03

**Authors:** Mutaz M Alenizi, Sultan D Alenazi, Sulaiman Almushir, Abdulkareem Alosaimi, Abdulkarim Alqarni, Irfan Anjum, Aamir Omair

**Affiliations:** 1 Internal Medicine, King Saud Bin Abdulaziz University for Health Sciences College of Medicine, Riyadh, SAU; 2 Clinical Psychology, King Saud Bin Abdulaziz University for Health Science, Riyadh, SAU; 3 Medical Education, King Saud Bin Abdulaziz University for Health Sciences College of Medicine, Riyadh, SAU

**Keywords:** prevalence, medical students, maladaptive daydreaming (md), generalized anxiety disorder (gad), grade point average (gpa)

## Abstract

This study demonstrates the prevalence of the maladaptive daydreaming (MD) and generalized anxiety disorder (GAD) among Saudi medical students. It also illustrates the association of MD with both GAD and the students’ grade point average (GPA). MD was assessed by using the 14-item Maladaptive Daydreaming Scale (MDS), and GAD was assessed by using both the 7-item Generalized Anxiety Disorder (GAD-7) questionnaire and the Penn State Worry Questionnaire (PSWQ). Our data estimate the prevalence of MD among the students to be 70%, GAD prevalence to be 80%, and 55% when using both GAD-7 and PSWQ, respectively. A statistically significant decline in the GPA was apparent among maladaptive daydreamers (MDers). A statistically significant rise in the scores of GAD-7 and PSWQ among MDers compared to non-MDers was found. To sum up, our findings demonstrate a high number of MDers and GAD sufferers among our subjects, and it appears that MD is associated with both GAD and GPA.

## Introduction

Daydreaming or spacing out is a temporary separation from reality during which people's contact with reality is compromised and somewhat replaced by a visionary fantasy that might be pleasant or annoying [1]. It is a normal cognitive phenomenon that happens to about 96% of Americans [2]. However, daydreaming might get serious and escalate to maladaptive daydreaming (MD) when people fail to provide adequate or appropriate adjustment to their environment or situation and dissociate from reality [2].

Although some people suffered from this condition, MD was not identified until it was first described by Professor Eliezer Somer [3]. MD can be defined as ‘‘an extensive fantasy activity that replaces human interaction and/or interferes with academic, interpersonal, or vocational functioning” [4]. Maladaptive daydreamers (MDers) spend hours indulging in highly structured and very immersive daydreams that are sometimes accompanied by repetitive movements, such as pacing and fidgeting [2]. Also, they have difficulties in shifting attention rather than sustaining attention, which is an inability to return to real life after being immersed in the fanciful daydreams [4]. Even though people with MD can have a variety of manifestations, it is not necessary for someone to have all of them to be considered an MDer. Common manifestations include daydreaming for lengthy periods, extremely vivid daydreams, difficulty completing everyday tasks, difficulty sleeping at night, an overwhelming desire to continue daydreaming, performing repetitive movements while daydreaming, showing some facial expressions while daydreaming, whispering and talking while daydreaming, and many other manifestations [4].

Many studies about MD have been carried out around the world, and the vast majority of them are recent because it is a relatively new condition that few researchers know about. One of the recent studies that were published in 2016 by Bigelsen et al. described MD and attempted to enhance the understanding of its features [4]. In that study, a total of 447 individuals, aged 13 - 78 years, from 45 countries were divided into two groups for the sake of comparison. Three hundred and forty were self-identified MDers and there were 107 controls. The results indicated that MD is completely different from normative daydreaming in many aspects and that a lot of MDers are having attention deficit, obsessive-compulsive, and dissociation symptoms [4]. Another study that was published in 2016 by Somer et al. described the development of the Maladaptive Daydreaming Scale (MDS), a 14-item self-report instrument designed to measure abnormal fantasizing [2]. The results showed that the instrument discriminated well between self-identified individuals with and without MD, and it seems to be an excellent measure for future investigation of MD. In a study published by Somer et al. in 2017, a new 16-item version of the MDS that included two additional items was used [5]. However, the study results showed high rates of comorbidities that accompany MD as 74% met criteria for more than three additional disorders, and 41% met criteria for more than four. Some of the most frequent MD comorbid disorders were obsessive-compulsive disorder, attention deficit hyperactivity disorder (ADHD), anxiety disorders, and depressive disorders. ­A recent MD research paper published in 2018 by Soffer-Dudek et al. showed a relationship between MD and obsessive-compulsive symptoms and possible shared mechanisms [6].

There is a growing number of MD studies; yet, it has not received much attention in Saudi Arabia. Currently, none of the published studies about MD were carried out in Saudi Arabia, and this means that there is a lot more to know about this condition in this region.

Recent articles have been written about MD, and it seems that researchers are now paying more attention to this phenomenon and trying their best to describe it. However, the etiology and the association of MD with other conditions are not clear. Some studies suggest that childhood stereotypic movement disorder could be associated with daydreaming; yet, what causes MD in the first place is still unknown [4]. Furthermore, future studies are needed to address the prevalence of MD in the general population. Additionally, although Somer et al. developed the MDS to determine if a person is an MDer or not, there are no guidelines recommending the application of this scale. Moreover, because certain MDers reported experiencing other disorders, such as ADHD, depression, schizophrenia, obsessive-compulsive disorder (OCD), and generalized anxiety disorder (GAD), it is reasonable to suspect that these disorders are related to MD but we still do not know how [6].

Schizophrenia might be misdiagnosed for MD because of overlapping symptoms, although schizophrenic patients cannot differentiate between the real and fictional worlds whereas MDers can [4]. Also, ADHD is similar to MD because in both conditions attention deficit and hyperactivity are found. ADHD involves disorganization, carelessness, and hyperactivity manifested as impatience and non-stop talking [7]. ­In addition, even though researchers found in one study that fluvoxamine (a drug that is a common treatment for OCD) was effective to control daydreams, there is no official treatment for MD yet [7]. The significance of doing more studies on MD lies in the fact that MD can interfere with people’s daily life, and it can be extremely difficult for MDers to get the help they need to deal with this condition. Wasting time on daydreaming can compromise functional, social, and academic life activities. In addition, there is a considerable number of MDers who reported a lack of concentration on their studies, impaired school performance, and skipping school just to be in their imaginary world [4]. Furthermore, many MDers with anxiety, worry, or depression reported that they escape from their difficult reality and cope with it by MD [8].

The aim of this research project was to explore and study MD in terms of its prevalence, its association with academic performance, and its relation to GAD. In particular, the specific objectives of this study were to estimate MD prevalence among the medical students in the College of Science and Health Professions at King Saud Bin Abdulaziz University for Health Sciences in Riyadh. Also, we aimed to identify whether there was an association between MD and GAD and to identify the possible association between the grade point average (GPA) of the students and both MD and GAD. Our plan is to describe MD by looking at this condition from different angles in the hope that this study might provide a comprehensive picture of MD.

## Materials and methods

This is an analytical cross-sectional study, and it was carried out during the academic year 2017 - 2018. The study was carried out in the College of Science and Health Professions (COSHP) at King Saud Bin Abdulaziz University for Health Sciences in Riyadh, Saudi Arabia. Approximately 1,204 students registered at the college during the academic year 2017 - 2018. All participants are Saudi undergraduate students, and most of them were between the ages of 18 to 21 years. In terms of inclusion criteria, all Saudi undergraduate students who study at the College of Science and Health Professions (COSHP) in Riyadh were included in the study, and nobody was excluded.

A total of 380 participants out of 1,204 Saudi undergraduate students who study at COSHP were selected and involved in the study. The sampling technique used in the study was the non-probability convenience sampling technique. Students who refused to participate in the study or did not complete the questionnaire were excluded from the study. The sample size was estimated by using the Raosoft® sample size calculator (http://www.raosoft.com/samplesize.html) with a confidence level of 95% and a 5% margin of error. In addition, as there was no reliable estimate of the prevalence of MD in the general population, the used response distribution was 50%. After performing the calculation, the optimal sample size was found to be 292 individuals. However, we added 30% to the sample size to ensure an adequate response rate and to compensate for the excluded students, resulting in a sample of 380 participants. Out of the 380 questionnaires, 306 completed questionnaires were received (response rate = 80.53%).

Data collection was carried out in the middle of the academic year by using self-administered questionnaires, and the authors distributed and collected the surveys from the subjects personally in the academic year 2017 - 2018 in COSHP. In this study, participants were asked to fill in their approximate GPA, as well as to complete three questionnaires. The first one was the MDS which was developed by Professor Somer et al., which included 14 items assessing some key characteristics of MD [2]. The items responses ranged on a scale from 0% to 100%, with 10% intervals (0% = never/none of the time; 100% = all of the time/extreme amounts). The MDS represents a highly reliable and valid new measure with a sensitivity of 95% and a specificity of 89% and can discriminate between MDers and normal individuals using a cut-off score of 25 out of 100 [2]. The second questionnaire was the GAD-7 which assessed GAD. The GAD-7 score is calculated by assigning scores of 0, 1, 2, and 3 to the response categories of 'not at all', 'several days', 'more than half the days', and 'nearly every day', respectively, and adding together the scores for the seven questions [9]. In GAD-7, the cut-off points for mild, moderate, and severe anxiety were the scores of 5, 10, and 15, respectively. GAD-7 can identify GAD with a sensitivity of 89% and a specificity of 82% using the threshold score of 10. The third questionnaire was the Penn State Worry Questionnaire (PSWQ), which is a 16-item questionnaire that measures the trait of worry [10]. Using a Likert rating from 1 (not at all typical of me) to 5 (very typical of me), the PSWQ can identify individuals with GAD. Additionally, PSWQ can differentiate patients with GAD from other anxiety disorders [11]. The instrument has a total range of 16 to 80, and by using a cut-off score of 45, PSWQ can provide a sensitivity of 99% and speciﬁcity of 98% for GAD [10].

The Statistical Package for Social Sciences (SPSS), version 24 (IBM SPSS Statistics, Armonk, NY) was used for data entry and analysis. Qualitative data like gender and academic performance (GPA), demonstrated as groups (excellent, best possible grade A (5.0 - 4.5); very good, next highest grade B (4.49 - 3.50); good, indicates average performance grade C (3.49 - 2.5); sufficient, lowest passing grade D (2.49 - 1.5); and insufficient, failing grade F (1.49 - 1.0), have been presented by frequencies and percentages. Numerical data (if needed), like age, have been presented by means and standard deviation.

A T-test was used to know whether there was a difference between MDers and non-MDers in terms of developing a GAD, as well as affecting academic performance (GPA). The T-test was also used to compare GAD patients with normal individuals in terms of affecting GPA. The analysis of variance (ANOVA) test was used to compare normal individuals with clinically positive GAD individuals (mild, moderate, severe anxiety) in terms of affecting GPA. A p-value of < 0.05 was considered to show a statistically significant difference/association for all of the statistical tests. Using a cut-off score of 25 (out of a maximum of 100) proved useful in our sample because it discriminated well between MDers and non-MDers. This mean score served as a reliable cut-off mark for the correct identification of MD given its superb sensitivity (95%) and high specificity (89%). The GAD-7 score is calculated by assigning scores of 0, 1, 2, and 3, to the response categories of 'not at all,' 'several days,' 'more than half the days,' and 'nearly every day,' respectively, and adding together the scores for the seven questions. Scores of 5, 10, and 15 are taken as the cut-off points for mild, moderate, and severe anxiety, respectively. When used as a screening tool, further evaluation was recommended when the score was 10 or greater. Using the threshold score of 10, the GAD-7 had a sensitivity of 89% and a specificity of 82% for GAD.

Participation in the research was voluntary, and refusal to participate in the research did not affect the participants in any way, shape, or form. Additionally, informed written consent was sought from all participants. During data collection, contact or demographic information was sought and participant identification was coded to ensure confidentiality and anonymity of participants. The research team used appropriate language, which was consistent with local cultural norms of respect and dignity. The authors aimed at minimizing any potential harm from the research, especially the effects of negative emotions. Since discussing MD and anxiety can be an emotionally-laden experience for participants, all participants were given the opportunity of a debriefing session with the researchers. In addition, the participants were given the opportunity to discuss the matter further with the principal investigator up to three months after the participation date regarding any emotional or psychological concerns in relation to the research. Finally, institutional review board (IRB) approval was sought and granted from King Abdullah International Medical Research Center.

## Results

Out of 380 students, 306 (81%) completed and returned the questionnaires. With a 0.64 standard deviation, the average (mean) GPA of the participants was 4.28 out of 5, the median was 4.44 out of 5, and the interquartile range was 0.7 (4.0 - 4.7). All participants were of Saudi background.

The prevalence of MD (MDS score ≥ 25) among the students (n = 215) was 70% (CI: 65% - 75%). The mean MDS score of the participants was 35.6 ± 18.8 (max = 100). In addition, the mean MDS score of participants who were classified as MDers (MDS score ≥ 25) was 44.9 ± 13.7. The prevalence of all levels of anxiety among the students (n = 245) was 80% (CI: 75% - 85%), whereas 20% (15% - 25%) were normal (n = 61). Among the 245 students who had anxiety (n = 133), 44% (n = 77) (CI: 39% - 49%) experienced mild anxiety, 25% (CI: 20% - 30%) experienced moderate anxiety, and 11% (n = 35) (CI: 6% - 16%) experienced severe anxiety. The mean GAD-7 score of the participants was 8.4 ± 4.5 (max = 21). By using the PSWQ, 55% (n = 169) (CI: 50% - 60%) were positive for having GAD. The mean PSWQ score of the participants was 47.4 ± 10.9 (max = 80) (Table 1).

**Table 1:** Prevalence CI: confidence intervals; GAD-7 groups: general anxiety disorder groups according to Generalized Anxiety Disorder 7-item (GAD-7) scale; MDS groups: maladaptive daydreaming groups according to Maladaptive Daydreaming Scale (MDS); N: number of individuals in the group; PSQW groups: general anxiety disorder groups according to Penn State Worry Questionnaire (PSWQ); %: percentage of individuals in each group out of the population

GAD-7 Groups	N	(%)	95% CI
Normal	61	20%	15-25%
Mild anxiety	133	43%	38-48%
Moderate anxiety	77	25%	20-30%
Severe anxiety	35	11%	6-16%
PSQW groups			
Normal	137	45%	40-50%
Positive	169	55%	50-60%
MDS groups			
Non-maladaptive daydreamer	91	30%	25-35%
Maladaptive daydreamer	215	70%	65-75%

**Figure 1:**
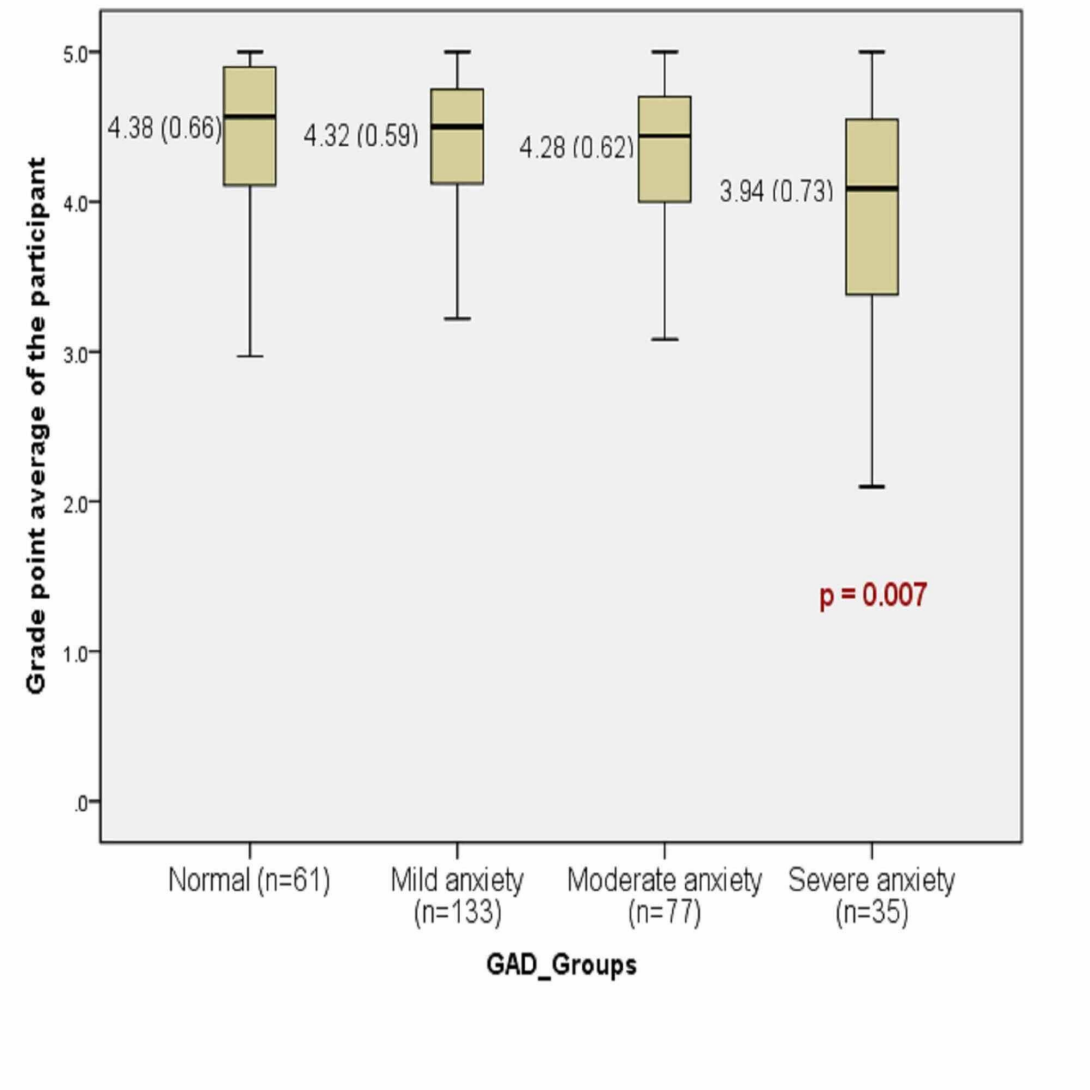
Generalized anxiety disorder 7-item (GAD-7) association with grade point average (GPA) GAD_Groups: General Anxiety Disorder groups according to GAD-7 scale

There was a statistically significant association between MD and GAD based on both GAD-7 and PSWQ questionnaires (p < 0.001) (Table 2). Also, MD has shown an association with academic performance (GPA) as the academic performance was significantly reduced among MDers (4.23 ± 0.66) compared to non-MDers (4.38 ± 0.56) (P = 0.035) (Table 2). Based on the PSWQ, the GPA was not significantly affected between GAD positive and normal individuals (Table 3). However, based on the subgroup classifications of the GAD-7 score, the academic performance (GPA) was significantly reduced among severely anxious individuals compared to the other groups (Table 4, Figure 1).

**Table 2:** MD Association with Grade Point Average (GPA) and Generalized Anxiety Disorder (GAD) GAD-7: Generalized Anxiety Disorder 7-item scale; MDers: maladaptive daydreamer; N: number of individuals in the group; Non-MDers: non-maladaptive daydreamers; PSQW: Penn State Worry Questionnaire

	MDers (N = 215)	Non-MDers (N = 91)	P-value
GPA	4.23 ± 0.66	4.38 ± 0.56	0.035
GAD-7	9.3 ± 4.6	6.1 ± 3.4	< 0.001
PSWQ	49.5 ± 11.2	42.7 ± 8.6	< 0.001

**Table 3:** General Anxiety Disorder (PSWQ) Association with Grade Point Average (GPA) N: number of individuals in the group; PSQW: Penn State Worry Questionnaire

		N	GPA	P-value
PSWQ	Positive	169	4.26 ± 0.61	0.73
	Normal	137	4.29 ± 0.67	

**Table 4:** Generalized Anxiety Disorder (GAD-7) Association with Grade Point Average (GPA) GAD-7: Generalized Anxiety Disorder 7-item scale; N: number of individuals in the group

		N	GPA	P-value
GAD-7	Normal	61	4.38 ± 0.66	0.007
	Mild anxiety	133	4.32 ± 0.59	
	Moderate anxiety	77	4.28 ± 0.62	
	Severe anxiety	35	3.94 ± 0.73	

## Discussion

This study demonstrates the prevalence of MD, as well as GAD, the relationship between the two, and their association with academic performance (GPA). Regarding the prevalence, it was surprising that 70% of the subjects were MDers, and no results about MD prevalence in the literature were found. In addition, the prevalence of GAD among the subjects in this study was found to be 80% by using the GAD-7 questionnaire and 55% by using PSWQ, and these results are different from the study conducted in King Faisal University, Saudi Arabia (14%) [12]. Also, these results are different from other studies conducted globally (7.3%) [13-14] and in other countries, such as Nigeria (0.1%), New Zealand (6.2%), and United States (3%) [15-16]. These results might be different from our study results because we used screening scales rather than diagnosing tools, which might explain the high prevalence of GAD among our population. In our study, students' academic performance has been observed to be influenced by a GAD, particularly among the severely anxious group compared to the other groups, based on the GAD-7 questionnaire classification. This result is supported by another study in which a community sample of children and adolescents were observed, and the results showed that anxiety is a common cause of poor academic performance among the students [17]. Similarly, another study supported the fact that anxiety contributes to poor academic achievement. However, their results cannot be compared with ours because different instruments were used to assess GAD and GPA [18]. In another study in which the relationship between test anxiety and academic performance among college students was investigated, there was no statistically significant association between test anxiety and educational performance. However, this result might be attributable to different educational environments [19].

One possible explanation for the high prevalence of both MD and GAD among the students is that they tend to predispose to one another. In other words, MD may develop as a means to escape from the harsh reality and anxiety into a safe fanciful world, and in the opposite way, a GAD can emerge due to the inability to adequately manage the time-consuming daydreams and the highly demanding academic obligations. Therefore, it can be viewed as a continuous cycle, and this can be supported by the fact that GAD tends to be a comorbid disorder to MD in a number of studies, including the Bigelsen et al. study where 7% of the participants reported anxiety [20], and Somer et al. study which showed that 28% of the participants had GAD [5]. Another possible explanation is that stress, anxiety, and mental health issues, in general, are far more common and can reach up to 64.3% and more in medical students compared to the general population, especially in the Middle East [21].

Some of the main limitations of our study were the non-randomized convenience sampling technique, and thus, our results cannot be generalized to a large population. Also, because it is a cross-sectional study, we do not know whether the association between the variables is causative or not. For example, it is not known whether severe anxiety affects academic performance or poor academic performance leads to severe anxiety. For this reason, further research is needed to address the association of nature. GAD-7 and PSWQ are not really diagnostic scales but are scales used in screening for anxiety disorders, and supplementing rating scales or clinical interviews should have been used in our study to provide more robust evidence. One of the reasons that the anxiety prevalence was so high was possible because the diagnosis of anxiety was solely based on GAD-7 and PSWQ which are screening tools designed to capture as many cases possible. Finally, the GPA was self-reported, and it is not considered as reliable as recorded college documents.

## Conclusions

This study makes an additional contribution to the tiny body of research on MD. We found that the majority of our subjects were MDers, and that MD is significantly associated with GAD among the students of the College of Science and Health Professions at King Saud Bin Abdulaziz University for Health Sciences in Riyadh, Saudi Arabia. We also found that GAD is significantly associated with GPA based on the GAD-7 questionnaire. In addition, MD was also significantly associated with GPA.
